# Supplementary material for: Integrating hypertension and HIV care in Namibia: A quality improvement collaborative approach
Source: PLoS One. 2022 Aug 11;17(8):e0272727. doi: 10.1371/journal.pone.0272727 (PMC9371294; doi:10.1371/journal.pone.0272727)
Supplement: Attachment — Submitted filename: Integrating hypertension and HIV care in Namibia.docx [file pone.0272727.s003.docx]

Integrating hypertension and HIV care in Namibia: a quality improvement collaborative approach

PONE-D-22-03989

In general, it is a little difficult to understand the processes of how to provide a quality improvement collaborative approach and how to reach the intervention package. The objective is not clear about the main outcomes to present.

Abstract

Page 2, Line 41: the term using routine measurement is not clear and could not find in the main method session.

Methods

Page 7, line 175: To capture “change ideas” and site-reported implementation barriers, who were interviewed?

Page 7 line 176: how many persons interviewed?

Page 7, line 180: How did all participating teams perform Plan-Do-Study-Act (PDSA) cycles? How did you get agreement for prioritizing for the intervention?

Page 7, line 181: If there is any change package that is out of domains of the Chronic Care

Model? If yes, how it handle?

Results

It would be better to explain a little bit more about Figure 1.

Methods

It would be better to explain how follow-up schedule is performed for HIV patients. Although it is a good

If hypertension screening is performed during the follow-up visit,

Page 4 ,line 114 Only 33% of facilities have physicians who are responsible person to prescribe the anti hypertension therapy. How can it be possible to manage all facilities to provide anti-hypertension for all patients every regular visit?

Page 5, line 125-138

It would be better to explain who will be involved in

Who

When

How

Table 2 What is the performance data?

In QI team meetings, who will involve and

Figure 3 2^nd^ Y axis should be HTN treatment rate.

How did they prioritize intervention? How do they get agreement for setting as the intervention? Who decides the intervention should be?

Page 7 line 175-176 How and who collected site-reported implementation barriers? Who reported these barriers? Who were interviewed for semi-structured interviews?
