# Supplementary material for: Integrating hypertension and HIV care in Namibia: A quality improvement collaborative approach
Source: PLoS One. 2022 Aug 11;17(8):e0272727. doi: 10.1371/journal.pone.0272727 (PMC9371294; doi:10.1371/journal.pone.0272727)
Supplement: Attachment — Submitted filename: PONE.docx [file pone.0272727.s005.docx]

PONE-D-22-03989R1: Integrating hypertension and HIV care in Namibia: a quality improvement collaborative

Approach

I agree with the previous responses. I only have some minor comments.

(1) In abstract, although task sharing was recommended in the conclusion, the information of onsite physician did not mention in the abstract methods and result.
