# Supplementary material for: Integrating hypertension and HIV care in Namibia: A quality improvement collaborative approach
Source: PLoS One. 2022 Aug 11;17(8):e0272727. doi: 10.1371/journal.pone.0272727 (PMC9371294; doi:10.1371/journal.pone.0272727)
Supplement: Attachment — Submitted filename: Response to Reviewers2 - Basenero et al..docx [file pone.0272727.s006.docx]

**Review Comments to the Author:**

Reviewer #1: Thank you for the hard work to improve the manuscript.

The authors selected study sites from only sites with high volume patients. The QIC approach may not work in areas with low volumes of patients. About this comment, the authors respond: “We agree with the reviewer that barriers and enablers to uptake of interventions in health systems are highly heterogeneous—and may vary according to patient volume. As a time-limited, donor-funded initiative, however, site selection was undertaken with the explicit aim of producing the greatest impact in the shortest period of time.” This limitation reduced the strength of the findings. I still believe that the finding of this study cannot be generalized to all of Namibia. The authors should remove all statements about integration of HTN and HIV services in Namibia. Policymakers and readers will misinterpret these findings. The authors should mention this limitation in the limitation section as well.

***We agree with the reviewer that scale up of the QIC to other sites in Namibia would require further adaptation, with explicit attention to how facilities with lower patient volumes might perform. In response to the reviewer’s comment, we have amended language in the Abstract (line 53) and Conclusion (line 322) to specify that integration of HTN and HIV services was promoted in the 24 sites participating in the QIC—and not Namibia as a whole. We have also amended language in the Limitations section (lines 308-310) to emphasize that the generalizability of the study’s results may be limited—particularly when considering patient volumes as the reviewer mentions.***

Reviewer #2: I agree with the previous responses. I only have ONE minor comment.

In the abstract, although task sharing was recommended in the conclusion, the information on on-site physicians was not mentioned in the abstract methods or results.

***In response to the reviewer’s comment, we have added language in the methods (lines 38-39) and results (lines 46-47) sections of the Abstract to report additional information regarding on-site physicians.***
