# Supplementary material for: Integrating hypertension and HIV care in Namibia: A quality improvement collaborative approach
Source: PLoS One. 2022 Aug 11;17(8):e0272727. doi: 10.1371/journal.pone.0272727 (PMC9371294; doi:10.1371/journal.pone.0272727)
Supplement: Attachment — Submitted filename: PONE_R2.docx [file pone.0272727.s007.docx]

PONE-D-22-03989R2

Integrating hypertension and HIV care in Namibia: a quality improvement collaborative approach

Thanks authors. I agree with your responses on previous comments. I do not have any comment on it.
